# Inactivation of the tumor suppressor gene *von Hippel-Lindau (VHL)* in granulocytes contributes to development of liver hemangiomas in a mouse model

**DOI:** 10.1186/s12885-016-2802-3

**Published:** 2016-10-12

**Authors:** Hannah L. Bader, Tien Hsu

**Affiliations:** 1 Department of Medicine, Boston University School of Medicine, Boston, MA USA; 2 Department of Biomedical Sciences and Engineering, National Central University, Chung-li, Taiwan

**Keywords:** von Hippel-Lindau, Hypoxia-inducible factor 2 alpha, Hemangioma, Placental growth factor, Extramedullary erythropoiesis, Hemangioblastoma, Neutrophil, Angiogenesis

## Abstract

**Background:**

Mutations in the tumor suppressor gene *von Hippel-Lindau (VHL)* underlie a hereditary cancer syndrome—VHL disease—and are also frequently observed in sporadic renal cell carcinoma of the clear cell type (ccRCC). VHL disease is characterized by malignant and benign tumors in a few specific tissues, including ccRCC, hemangioblastoma and pheochromocytoma. The etiology of these tumors remains unresolved.

**Methods:**

Conditional inactivation of the *VHL* gene in mouse (*Vhlh*) was generated to examine the pathophysiological role of the *VHL* gene function. Specific cell populations were isolated by fluorescence-activated cell sorting (FACS) and bone marrow transplants were performed to identify the *Vhlh*-inactivated cells responsible for the phenotype.

**Results:**

Previously we showed that inactivation of *Vhlh* in a subpopulation of kidney distal tubule cells resulted in hyperplastic clear-cell lesions and severe inflammation and fibrosis. Here, we show that this knockout mouse strain also develops *Hif-2α*-dependent vascular overgrowth (hemangioma) and extramedullary erythropoiesis in the liver. However, *Vhlh* inactivation was not detected in the liver parenchyma. We instead demonstrate that in these mice, *Vhlh* is inactivated in liver granulocytes and that hemangiomas are partially rescued in knockout mice reconstituted with wild-type hematopoietic stem cells, indicating the involvement of bone-marrow-derived leukocyte. Interestingly, bone marrow from knockout mice failed to generate the liver phenotype in wild-type recipients, suggesting that an additional cell type that is not derived from the bone marrow is involved in the development of the hemangioma phenotype.

**Conclusion:**

These results support the idea that the development of a full-blown VHL disease phenotype requires inactivation of the *VHL* gene not only in the tumor proper, but also in the stromal compartment.

## Background

Patients with VHL disease are heterozygous for *VHL* mutations, and develop tumors when the function of the remaining wild-type *VHL* allele is lost via somatic mutation or epigenetic silencing [1]. VHL tumors, which can occur in several different tissues, are characterized by hypervascularity and a clear cell appearance in histological preparations. *VHL* mutations are also frequently observed in sporadic renal cell carcinoma (ccRCC). In addition, specific *VHL* missense mutations have been described that do not cause tumors, but result instead in recessive polycythemia, a disease characterized by an overproduction of erythrocytes [2–4].

VHL protein (pVHL) is an essential negative regulator of the hypoxia-inducible factor (HIF), a transcription factor induced by low oxygen tension [5]. HIF induces a metabolic switch from oxidative phosphorylation to glycolysis, which is essential for cell survival under hypoxic conditions. HIF also promotes angiogenesis and erythropoiesis through induction of cytokines such as vascular endothelial growth factor (VEGF) and erythropoietin (EPO). The active HIF transcription factor is a dimer consisting of an α and a β subunit [1, 5]. The β unit—known as HIF-1β or ARNT (arylcarbon receptor nuclear translocator)—is ubiquitously and constitutively expressed. In contrast, the HIF-α subunits (HIF-1α, HIF-2α and HIF-3α) are regulated by oxygen tension. Under normoxic conditions, HIF-α is hydroxylated. The hydroxylated form is recognized by an ubiquitin ligase and undergoes ubiquitination, followed by proteasome-mediated degradation. Hydroxylation is oxygen dependent, and is inhibited under hypoxic conditions. Thus, hypoxia leads to stabilization of the HIF-α protein, allowing formation of the dimeric HIF transcription factor and transactivation (or repression) of HIF responsive genes.

pVHL is the substrate recognition component of the multimeric ubiquitin-ligase complex that mediates HIF-α ubiquitination [1, 5]. *VHL* gene inactivation therefore leads to normoxic stabilization of HIF-α and inappropriate activation of the HIF transcription factor. The formation of VHL tumors is thought to be driven in large part by genes induced or suppressed by HIF [5]. However, loss of *HIF*-independent functions of *VHL*, and mutations of additional tumor suppressor genes, likely also contribute to tumorigenesis [1, 6, 7]. Recent animal model and cancer genome studies have indicated that *VHL* mutations are necessary but insufficient for tumorigenesis [6, 8–10]. Such second and even third hits conceivably can be additional genetic or epigenetic changes within the same cells, or can be within a separate cell population that contributes to the formation of tumor microenvironment. The requirement for additional tumor suppressor gene(s) in ccRCC formation was supported by the construction of *Vhlh* (mouse allele of *VHL*)*-Bap1* double knockout [11]. *BAP1* gene mutations have been observed in ~10 % of ccRCC samples [9, 10]. *Vhlh-Bap1* double knockout generated clear-cell lesions that resemble carcinoma [11]. On the other hand, mutations in the cancer stromal cells, including those of the well-known tumor suppressor genes *p53* and *PTEN*, have been documented that contribute to cancer progression {reviewed in [12]}. It is therefore possible that in VHL patients, *VHL* inactivation could also occur in the tumor microenvironment (stroma) in addition to the tumor itself.

One of the most frequently observed tumors in VHL patients besides ccRCC is hemangioblastoma, a highly vascularized tumor with extramedullary hematopoiesis that occurs in the central nervous system and the retina [13]. Hemangioblastomas cause considerable morbidity and mortality despite being benign. Hemangioblastomas are sometimes referred to as vascular tumors; however, biallelic inactivation of *VHL* was detected in the stromal compartment of the vascular tumors [14–16], which also have a clear cell appearance. Vascular overgrowth is therefore likely induced by pro-angiogenic cytokines released by these “stromal cells.” In addition, hemangioblastomas frequently contain foci of extramedullary erythropoiesis and the *VHL* ^*−*^ stromal cells exhibit multipotency that may be of embryonic origin [17–19]. There are no mouse models that recapitulate hemangioblastoma. However, several VHL mouse models develop hemangiomas—an overgrowth of irregularly shaped and leaky blood vessels—in the liver [20–23]. Hemangiomas have been observed in the liver of germline *Vhlh* ^+/-^ mice [20] and in mosaic *Vhlh* biallelic deletion mice induced by conditional *β-actin*-driven *Cre* [21]. These two models contain heterozygous and homozygous, respectively, *Vhlh* mutants in most cell types, including hepatocytes and endothelial cells. More interestingly, liver hemangiomas were also observed in *PEPCK-Cre* driven *Vhlh* knockout, which inactivates *Vhlh* in renal proximal tubule cells and in ~20 to 30 % of hepatocytes [20, 22]. Likely due to early mortality, full-blown hemangiomas were not observed when a more hepatocyte-specific *Cre* driver, *Albumin-Cre*, was used to inactivate *Vhlh*; nonetheless, numerous blood-filled vascular cavities, and foci of increased vascularization within the hepatic parenchyma were observed [20, 22]. Inactivation of *Vhlh* in hepatocytes with *PEPCK-Cre* or *Albumin-Cre* also led to erythrocytosis—overproduction of erythrocytes—due to increased expression of Epo [20, 22], although hemangioma-associated extramedullary erythropoiesis—as observed in hemangioblastoma—was not observed. Hif-2α was found to mediate up-regulation of erythropoietin and multiple pro-angiogenic cytokines in these mouse models, and inactivation of *Hif-2α* or *Hif-1β/Arnt,* but not *Hif-1α,* rescued hemangiomas in *PEPCK-Cre or Albumin-Cre* driven *Vhlh* knockout mice [22, 24].

Previously we showed that inactivation of *Vhlh* in a subpopulation of kidney distal tubules, using the *HOXB7-Cre* driver, resulted in *Hif-1α*-dependent hyperplastic clear-cell lesions and severe inflammation and fibrosis [25]. Here, we report that the same *HOXB7-Cre* driven *Vhlh* conditional knockout mice also developed liver hemangiomas as well as extramedullary erythropoiesis. Interestingly, in contrast to the previous mouse models, we did not detect *Vhlh* inactivation in hepatocytes. Instead, *Vhlh* inactivation was detected in liver granulocytes in the knockout mice. In support of a myeloid component in the development of hemangiomas in the liver, reconstitution of the knockout mice with wild-type hematopoietic stem cells partially rescued the hemangioma phenotype. Further analysis showed that the granulocyte population contained the *Vhlh* deleted allele. In addition, granulocytes (neutrophils) in livers of the *HOXB7-Cre* driven *Vhlh* knockout mice were found to over-express placental growth factor (PlGF) that has been shown to promote angiogenesis. Thus, this mouse model supports the notion that a bone marrow-derived stromal component with *VHL* loss-of-function contributes to the development of the full extent of the VHL disease phenotype.

## Methods

### Animal protocol and mouse strains

All of the procedures were conducted in accordance with the US Public Health Service Policy on Humane Care and Use of Laboratory Animals. Mice used in these studies were maintained in Boston University Medical Center facility according to protocols approved by the Institutional Animal Care and Use Committee. Mouse strains used were in C57BL/6 background and have been described previously [25]. For generation of bone marrow chimeras, B6.SJL-*Ptprc* ^*a*^ *Pepc* ^*b*^/BoyJ (“B6 CD45.1”) as well as RosaLacZ was purchased from Jackson Laboratories (Bar Harbor, Maine, USA).

### Reagents

Phosphate-buffered saline (PBS), Dulbecco’s phosphate-buffered saline (DPBS), Dulbecco’s modified eagle medium (DMEM) and HEPES were obtained from Gibco/Life Technologies (Carlsbad, CA, USA). Fetal bovine serum (FBS) was obtained from Hyclone (Logan, UT, USA). Sterile 0.5 M EDTA stock solution, pH7.5, was obtained from Boston Bioproducts (Ashland, MA, USA). Fluorescence-activated cell sorting (FACS) buffer was prepared as follows: 0.5 % FBS/2 mM EDTA in DPBS. Red blood cell lysis buffer, Fc-block and antibodies for flow cytometry (except anti-CD45 antibody) were obtained from eBioscience (San Diego, CA, USA). Cell strainers (40 μm or 70 μm) were obtained from ThermoFisher Scientific (Waltham, MA, USA).

### Histology and immunohistochemistry

Livers were fixed overnight in 10 % neutral buffered formalin and were embedded in paraffin. 4 μm thick paraffin sections were stained with hematoxylin and eosin (H&E) according to standard procedures. For immunohistochemistry, 4 μm thick paraffin sections were dewaxed, and heat mediated antigen retrieval was performed with citrate buffer, pH 6, for 40 min. Endogenous peroxidase was quenched by incubating sections for 15 min in methanol with 0.3 % H_2_O_2_ or peroxidase block (Peroxidased 1, Biocare Medical, Concord, CA). Endogenous biotin was blocked with avidin-biotin blocking kit (Vector Laboratories, Burlingame, CA, USA), followed by 30 min blocking with 3 % or 10 % (GFP stain) normal goat serum (Sigma-Aldrich, St. Louis, MO) in PBS. Staining and washing was performed with PBS, 0.05 % Tween 20 (Sigma-Aldrich). Sections were incubated overnight at 4 °C with primary antibody (1:50 rat anti-CD45, clone 30-F11, Molecular Probes/Life Technologies, Carlsbad, CA, USA; 1:100 rabbit anti-mouse Plgf, Origene/Acris Antibodies, Rockville, MD, USA; 1/500 chicken anti-GFP, Abcam) and incubated for 45 min with appropriate biotinylated secondary antibody (Vector Laboratories) at 1/500 (rabbit and chick secondary) or 1/1000 (rat secondary). After washing 3 × 5 min (CD45) or 4 × 15 min [placental growth factor Plgf), GFP], sections were incubated for 45 min with streptavidin-conjugated horseradish peroxidase (Invitrogen/Zymed, Carlsbad, CA, USA) at 1/1000 (CD45 stain) or with ABC Elite Kit (Vector Laboratories; Plgf, GFP stain). Sections were washed again for 3 × 5 min (CD45) or 4 × 15 min (Plgf, GFP) and were incubated for 5–10 min with peroxidase substrate (DAB, Vector Laboratories). Sections were counterstained with hematoxyline and mounted with permount (ThermoFisher Scientific).

### Preparation of single cell suspensions from liver

Livers were dissected out taking care to minimize bleeding, and were rinsed with DPBS to wash off excess blood. For wild-type samples, small pieces of liver were dissected out from the left and median lobe (lobe encasing the gallbladder). For knockout samples, liver pieces containing hemangiomas were dissected out. In some cases hemangioma tissue was pooled from two mice to obtain enough material. Next, liver tissue was minced and resuspended in 5–10 ml digestion buffer consisting of ice-cold DMEM with 5 mg/ml (or 800 U/ml) collagenase (trypsin-free Collagenase, CLS-4, Worthington Biochemical Corporation, Lakewood, NY, USA) and 20 mM HEPES. Digestion was performed at 4 °C for 1–1.5 h in 5-ml round bottom polypropylene tubes with overhead rotation. The digest was then diluted 2-fold in FACS buffer, EDTA was added to a final concentration of 2 mM, and the cell solution was strained through a 70-μm strainer. Next, cells were pelleted at 250 xg for 8 min at 4 °C, and resuspended in ice-cold red blood cell lysis buffer (from eBioscience, San Diego, CA, USA). Red blood cell lysis was performed for 3 min on ice. After washing, liver cells were resuspended in FACS buffer and stained as described below.

### Flow cytometry and FACS

Staining was carried out in 1.5-ml tubes. For wash steps, cells were pelleted in a tabletop centrifuge (250 xg at 4 °C for 5 min). After treatment with red blood cell lysis buffer (see preparation of single cell suspensions), cells were resuspended in ice-cold FACS buffer and concentration was adjusted to 1 × 10^6^ cells–5 × 10^6^ cells per 100 μl. Cells were incubated with Fc-block (1:100) for 5 min on ice, before adding primary antibodies. After cells were incubated for 20 min on ice with primary antibodies, live/dead stain was performed. For propidium iodide staining, cells were washed once with 1 ml FACS buffer, and resuspended in FACS buffer with 1 μg/ml propidium iodide (1 mg/ml stock solution obtained from Life Technologies). Cells were then transferred to round bottom polypropylene tubes for sorting/analysis (no washing required after propidium iodide step). For staining with Aqua Blue Live/Dead solution (Life Technologies), cells were resuspended in 1 ml FACS buffer with 1:1000 of Aqua Blue stock solution (Life Technologies; stock solution prepared according to instructions of manufacturer) and were incubated for 15 min at 4 °C with overhead rotation (washing after antibody staining and live/dead staining in one step). Subsequently, cells were washed once with 1 ml FACS buffer, and were resuspended in FACS buffer and transferred to round bottom polypropylene tubes for sorting/analysis; cell suspensions were filtered through a 40-μm strainer. For sorting of CD45+ cells, cells were stained with CD45-APC (1:200; Molecular Probes/Life Technologies), followed by propidium iodide stain (Life Technologies). For quantification and sorting of erythrocyte progenitors, cells were triple stained with the following antibodies: CD45-Percp-Cy5 1:200, TER119-APC 1:100 and CD71-PE 1:200; followed by Aqua Blue Live/Dead stain (Life Technologies). For determination of chimerism in peripheral blood, cells were double-stained with CD45.1-PE and CD45.2-APC at 1:100 and dead cells were gated out according to size (FSC vs SSC plots). Cell sorting and analysis of liver samples was performed with the FACS Aria II SORP (Becton-Dickinson) or Beckman-Coulter Moflo.

### Liver colony forming unit assay

Livers were dissected out taking care to minimize bleeding, and were rinsed with 20 ml DPBS to rinse off excess blood. Subsequently, homogenization was performed as described above but without collagenase treatment. After passing the cell suspension through a 70-μm strainer, cells were pelleted (250 xg at 4 °C for 8 min) and resuspended in 20 ml DPBS containing 2 % FBS. For cell counting, a small aliquot was stained with an acridine orange and propidium iodide solution (AO/PI solution, Nexcelcom, Lawrence, MA, USA) according to the instructions of the manufacturer, and counted with the cellometer (Nexcelcom). 4.8 × 10^5^ live cells in 300 μl DPBS were then added to 3 ml of MethoCult GF M3434 (Stem Cell Technologies, Vancouver, BC, Canada) and plated out into two 3-cm tissue culture dishes, following instructions of the manufacturer. BFU-E colonies per plate were quantified in a blinded fashion after 7–8 days, and were averaged for each sample (2 plates per sample).

### β-galactosidase staining of organs

Chemicals for staining were obtained from ThermoFisher Scientific. Livers and kidneys were fixed in 4 % paraformaldehyde in PBS for ~4 h at room temperature. Organs were then washed (3 × 30 min) with wash buffer (0.1 M NaH_2_PO_4_, 0.1 M Na_2_HPO_4_, 2 mM MgCl_2_, 0.01 % deoxycholate, 0.02 % NP-40) and stained overnight at 4 °C with staining buffer (wash buffer with 5 mM ferrocyanide, 5 mM ferricyanide and 1 mg/ml 5-bromo-4-chloro-3-indolyl-β-D-galactopyranoside (X-Gal). Organs were photographed, post-fixed for 15 min with 4 % paraformaldehyde in PBS, and were embedded in paraffin. 4 μm thick paraffin sections were prepared and counter-stained with Nuclear Fast Red (Vector Laboratories) and analyzed for β-galactosidase staining.

### Isolation of genomic DNA and polymerase chain reaction (PCR) for detecting *Vhlh* deletion

DNA from liver tissue was obtained using the DNAeasy blood and tissue kit (Qiagen, Valencia, CA, USA) according to instructions of the manufacturer. Sorted cells (10,000-500,000) were pelleted in a table top centrifuge (300 xg, 5 min), frozen in <100 μl DPBS with 2 % FBS, and stored at −80 °C. Subsequently, DNA was obtained with the Qiamp Micro DNA kit (Qiagen) following the protocol for DNA isolation from small amounts of blood. *Vhlh* primers for detection of the *Vhlh* flox allele and wild-type allele have been described before [22]; sequences are as follows: *Vhlh-wt/flox* forward primer (FW1), ctaggcaccgagcttagaggtttgcg; *Vhlh-wt/flox* reverse primer (Rev1), ctgacttccactgatgcttgtcacag. PCR products are ~290 bp (*wt* allele) and 460 bp (*floxed* allele). The site of the forward primer is lost upon recombination; the *Vhlh-wt/flox* primers therefore cannot amplify the recombined/deleted *Vhlh* allele. Generic primers were used to detect Cre: *FW*, atccgaaaagaaaacgttga; *Rev*, atccaggttacggatatagt; Cre-PCR product is ~700 bp. *Vhlh* deletion primers were designed as follows: the forward primer (FW2) is situated downstream of the second HindIII restriction site, and upstream of the NdeI restriction site within the 5′ untranslated sequence of the murine *Vhlh* gene. The reverse primer (Rev2) is situated downstream of the HindIII restriction site within the first intron of the murine *Vhlh* gene [20]. The sequences are as follows: *Vhlh-del* forward primer (FW2): ggaaccatctcttctctgatagagc; *Vhlh–del* reverse primer (Rev2): gctggttgcttcagacacaatcttg. The *Vhlh del* primers flank exon 1 (see Fig. 4c). In the presence of exon 1, the sequence is very long (~4 kb) and is therefore not amplified under stringent PCR conditions (e.g., short extension time). In the presence of Cre, exon 1 is excised, resulting in a much shorter sequence, allowing amplification of the recombined/deleted *Vhlh* allele. The *Vhlh-del* PCR product is ~ 800 bp. Identity of the product was confirmed by sequencing.

### Serum collection and ELISA for erythropoietin

Peripheral blood was collected in heparinized microcapillaries (ThermoFisher Scientific), and was transferred into collection tubes with clotting activator (BD Microtainer SST, Becton Dickinson, Waltham, MA, USA). Blood was incubated for ~5–10 min at room temperature, and centrifuged at full speed in a table-top centrifuge. The serum (corresponding to supernatant) was collected and stored at −80 °C for up to 4 months. Subsequently, erythropoietin ELISA was performed with Quantikine erythropoietin ELISA kit (RD systems Inc, Minneapolis, MN, USA) according to instructions of the manufacturer.

### Preparation of cDNA and quantitative PCR

Liver RNA was isolated using Trizol in combination with Purelink RNA Mini kit (Invitrogen/Life Technologies) according to instructions of manufacturer. DNA was digested with on-column DNAse I digestion kit (Invitrogen/Life Technologies) according to instructions of manufacturer. Sorted cells (~20,000) were pelleted, resuspended in Quiazol (Qiagen) and stored at −80 °C. Subsequently, RNA was purified using the miRNAeasy Kit (Qiagen) according to instructions of the manufacturer. After elution, RNA from sorted cells was dried in GenTegra™-RNA tubes (GenTegra, Pleasanton, CA, USA) and was resuspended in ~5 μl of water. Liver RNA (1 μg) or entire RNA obtained from ~20,000 sorted cells was reverse transcribed with AMV First Strand cDNA kit (liver) or Protoscript II first strand synthesis kit (sorted cells; both kits from New England Biolabs, Ipswich, MA, USA) according to instructions of the manufacturer. Real-time PCR was performed with Power SYBR Green Mastermix (Applied Biosystems/Life Technologies) using a StepOne Real time PCR system (Applied Biosystems/Life Technologies). The following primers from the Universal Probe Library (Roche/Life Technologies) were used: 18 s forward primer, gcaattattccccatgaacg; 18 s reverse primer, gggacttaatcaacgcaagc; erythropoietin forward primer, ccctgctgcttttactctcc; erythropoietin reverse primer, gggggagcacagaggact; prolyl-hydroxylase 3 (Phd3) forward primer, tgtctggtacttcgatgctga; reverse primer, agcaagagcagattcagtttttc.

### Hoechst staining and bone marrow transplant

Staining with Hoechst 33342 (Life Technologies) was performed as described previously [26, 27], with the following modification: to increase the yield, Hoechst was titrated so that cells were understained (to achieve a Hoechst negative side population of ~1 %). Subsequently, only the least Hoechst positive cells (comprising 0.2–0.5 % of all cells) were sorted, and 1000 SP were used per recipient; 3 × 10^5^ unfractionated bone marrow cells were co-transplanted to improve survival. Recipients were lethally irradiated one day before transplantation with a split dose of 14 gray (2 month old recipients) or 11 gray (4 week old recipients) separated by 2 h. For 2 weeks after the transplant, starting with the day of the transplant, recipients received antibiotics in the drinking water. To examine chimerism using flow cytometry, peripheral blood was collected in heparinized microcapillaries (ThermoFisher Scientific). Blood samples (~100 μl) were then transferred to 1.5 ml tubes containing 100 μl DPBS with 2 mM EDTA. 1 ml of red blood cell lysis buffer was added to samples, and red blood cell lysis was carried out 5 min at room temperature. After adding 10 ml of ice cold FACS-buffer, samples were transferred to 15 ml centrifuge tubes and were centrifuged (8 min, 250 g, 4 °C). Cells were then resuspended in 100 μl FACS-buffer, and stained with antibodies for flow cytometry (see above).

### Statistical analysis

Unpaired, two-tailed *t*-tests were performed; where necessary, Welch’s correction for unequal variances was applied. All analyses were performed using GraphPad Prism Software (La Jolla, CA, USA). For group comparisons, *p*-values were calculated with GraphPad prism software, and false discovery rate (FDR) was calculated manually using Bonferroni post-test or Benjamini and Hochberg FDR formula.

## Results

### Hepatic hemangioma phenotype in the *HOXB7-Cre* driven *Vhlh* knockout mice

*HOXB7-Cre* is widely used to target the collecting ducts of the kidney [28, 29]. However, we recently found that this *Cre* driver is also expressed in a subset of distal tubules in the kidney cortex. Conditional inactivation of *Vhlh* using this *Cre* driver resulted in fully penetrant hyperplasia, cysts, clear-cell lesions, inflammation and fibrosis in the kidney, with the hyperplastic lesions arising primarily from Tamm-Horsfall positive distal tubules [25].

**Fig. 1:**
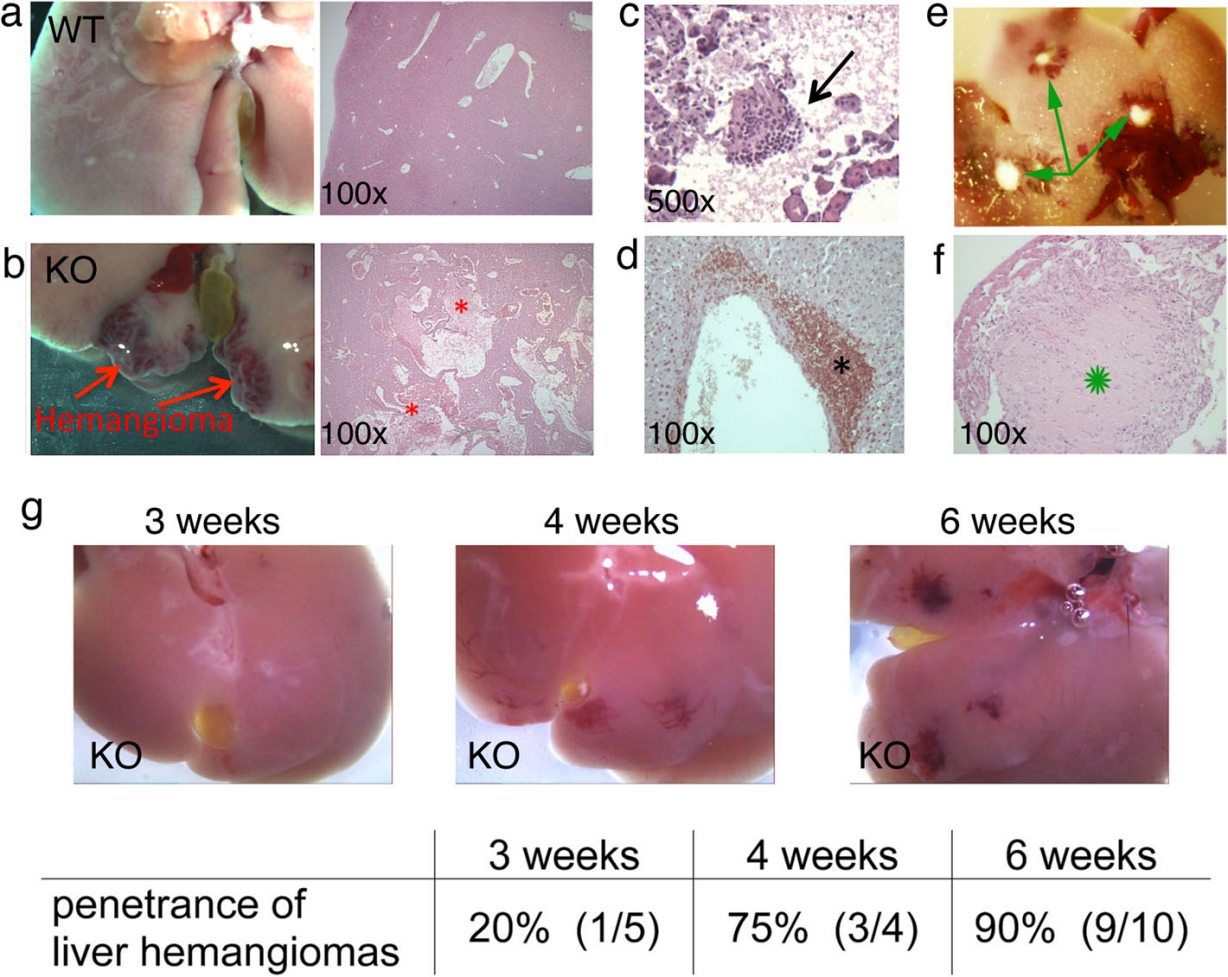
*HOXB7-Cre; Vhlh* ^*fl/fl*^ mice develop hemangiomas and fibrotic lesions in the liver. **a** Whole-mount (*left*) and H&E-stained section (*right*) of wild-type mouse liver from a *HOXB7-Cre; Vhlh* ^*+/+*^ mouse. **b** Whole-mount (*left*) and H&E-stained section (*right*) of knockout mouse liver from a *HOXB7-Cre; Vhlh* ^*fl/fl*^ mouse. Hemangiomas are indicated by arrows and erythrocyte-filled blood vessels are indicated by asterisks (*; interpreted by a pathologist). **c** H&E-stained liver section from a *HOXB7-Cre; Vhlh* ^*fl/fl*^ mouse. A cluster of leukocytes within the hemangioma is indicated by an arrow (interpreted by a pathologist). **d** Liver section from a *HOXB7-Cre; Vhlh* ^*fl/fl*^ knockout mouse, stained for CD45 (a pan-leukocyte marker). A cluster of leukocytes near the blood vessel is indicated (*; interpreted by a pathologist). **e** Whole-mount of liver from a *HOXB7-Cre; Vhlh* ^*fl/fl*^ knockout mouse. Fibrotic foci are indicated by arrows. **f** H&E-stained section of liver from a *HOXB7-Cre; Vhlh* ^*fl/fl*^ knockout mouse. A large fibrotic site is indicated (*; interpreted by a pathologist). **g** Representative images of whole-mounted livers of 3, 4 and 6-week old *HOXB7-Cre; Vhlh* ^*fl/fl*^ mice. The penetrance of hemangiomas at 3, 4, and 6 weeks of age is indicated in the table below the images. Numbers in brackets refer to mice with hemangiomas versus total mice analyzed (n with hemangiomas/n total)

Interestingly, these *HOXB7-Cre*; *Vhlh* ^*fl/fl*^ mice also developed hemangiomas in the liver starting at 3 weeks of age. The diseased liver showed an overgrowth of irregularly shaped, abnormally large and leaky blood vessels (compare Fig. 1a, b). Erythrocytes (Fig. 1b) and leukocytes (Fig. 1c) accumulated within the hemangiomas, based on pathologist’s assessment. Leukocytes were also identified adjacent to hemangiomas by immunohistochemistry for CD45-expressing cells (Fig. 1d; based on pathologist’s assessment). Older mice (>3 months) additionally developed fibrotic lesions (Fig. 1e, f), indicating involvement of an immune component. The development of hemangioma starts between 3 and 4 weeks of age and the penetrance reached 90 % at 6 weeks (Fig. 1g). In contrast, the development of kidney lesions in this knockout mouse strain begins at 8 weeks and becomes fully penetrant at 12 weeks [25].

### Extramedullary erythropoiesis in the liver of *HOXB7-Cre* driven *Vhlh* knockout mice

**Fig. 2:**
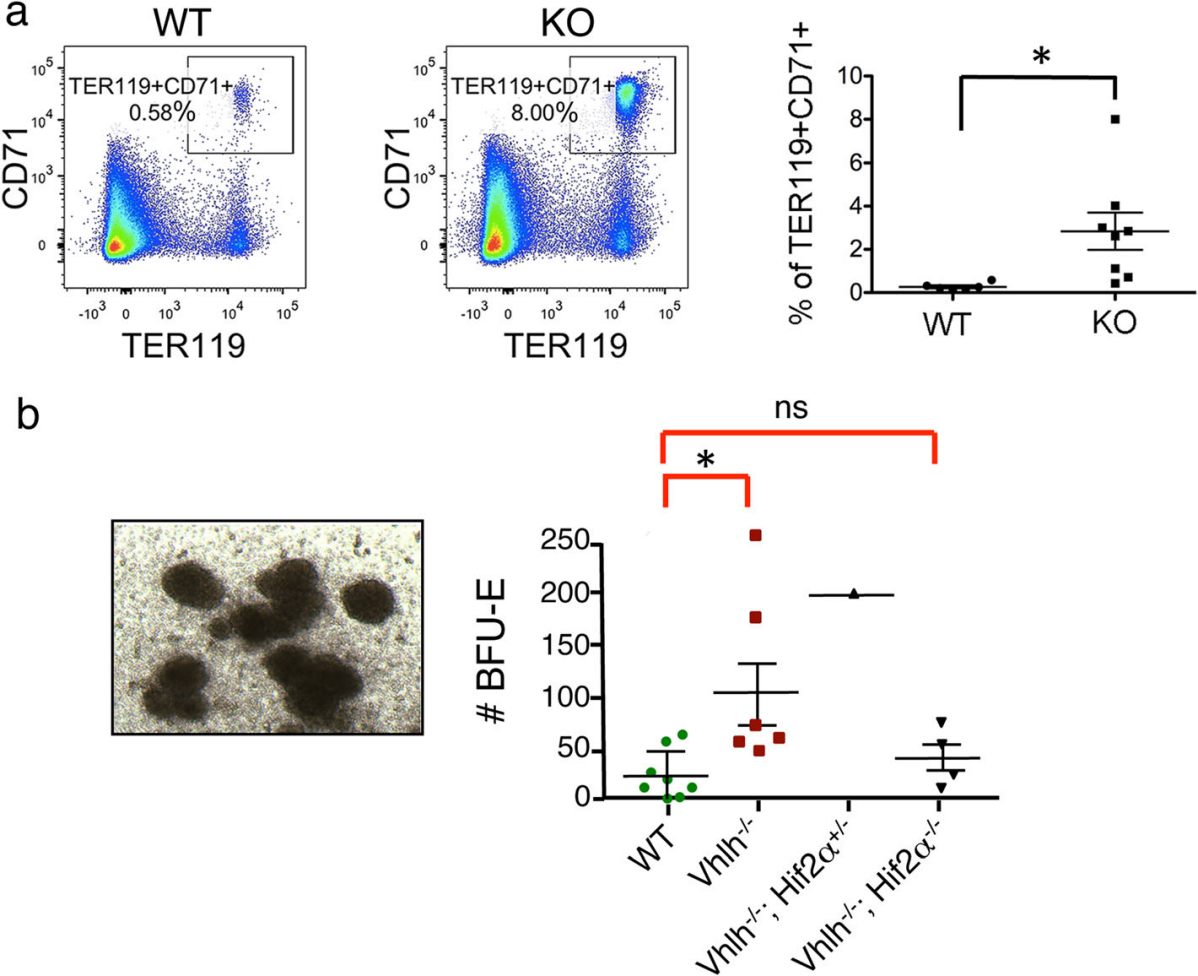
Extramedullary erythropoiesis in *Vhlh* knockout mice. **a** Erythrocyte progenitors are increased by ~10-fold in livers of *HOXB7-Cre; Vhlh* ^*fl/fl*^ knockout mice. Liver cell suspensions were prepared by mincing livers, followed by collagenase digestion and treatment with red blood cell lysis buffer (see Methods). Flow cytometric quantification of erythrocyte progenitors (TER119 + CD71+) was performed. Shown are representative FACS-plots (*left panels*) and quantification (*right panel*). Gate was set on live CD45+ cells, and doublets were gated out. **b** Quantification of erythroid progenitors with colony forming unit assay. Liver cell suspensions from *HOXB7-Cre; Vhlh* ^*fl/fl*^ (Vhlh^-/-^), *HOXB7-Cre; Vhlh* ^*fl/fl*^ *; Hif2α* ^*fl/+*^ (Vhlh^-/-^; Hif2α^+/-^), *HOXB7-Cre; Vhlh* ^*fl/fl*^ *; Hif2α* ^*fl/fl*^ (Vhlh^-/-^; Hif2α^-/-^), and *Vhlh* ^*fl/fl*^ (WT) mice were prepared and cultured as described in Methods. Left panel: Representative image of erythroid progenitor colonies (BFU-E) from a *Vhlh* knockout mouse. Right panel: Quantification of BFU-Es. While *Vhlh* knockout increased the number of BFU-E compared to that obtained from liver cell suspensions of *Cre negative* mice (WT), no difference was seen in the number of BFU-Es from WT and *HOXB7-Cre Vhlh-Hif-2α* double knockouts. One *Vhlh* ^*-/-*^ and *Hif-2α* single allele knockout animal of the genotype *HOXB7-Cre; Vhlh* ^*fl/fl*^ *; Hif-2α* ^*fl/+*^ (Vhlh^-/-^; Hif2α^+/-^) was used as a reference, which showed increased BFU-E number as in the *Vhlh* knockout. Mice were >3 months old. p-values were calculated using Student’s *t*-test with Welch’s correction. *: statistically significant (*p* < 0.05); ns: not significant

**Fig. 3:**
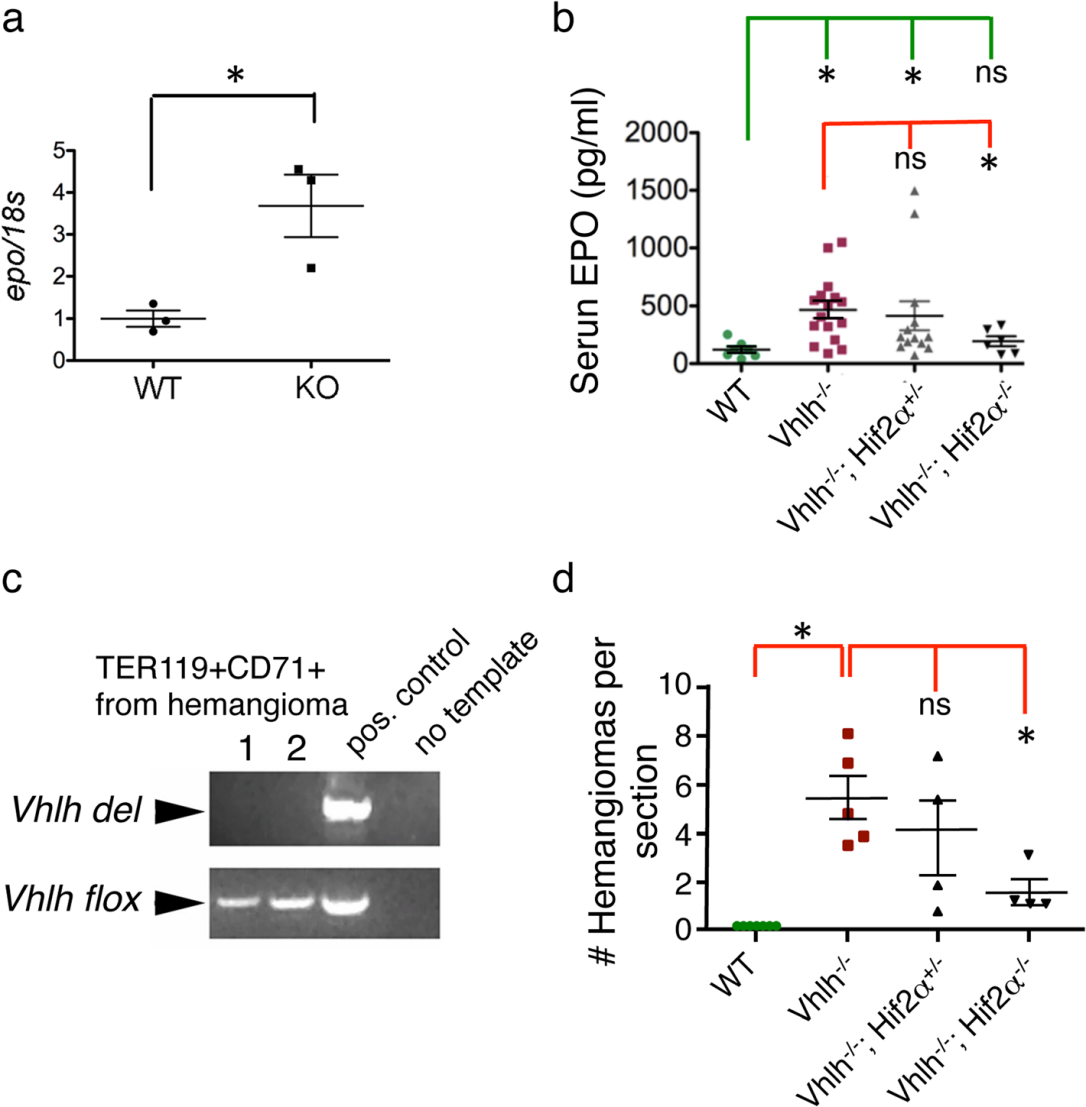
Inactivation of *Hif-2α* rescues extramedullary erythropoiesis and suppresses hemangiomas. **a** Erythropoietin is over-expressed in livers of *HOXB7-Cre; Vhlh* ^*fl/fl*^ knockout mice: analysis of *Erythropoietin (Epo)* mRNA expression by quantitative PCR. **b** Serum erythropoietin levels were determined by ELISA for indicated genotypes. Data were pooled from mice of >2 months of age. Erythropoietin was elevated by ~5 folds in the serum of *HOXB7-Cre; Vhlh* ^*fl/fl*^ (Vhlh^-/-^) mice compared to *HOXB7-Cre; Vhlh* ^*+/+*^ or Cre negative (WT) littermates. Upon *Hif-2α* inactivation (*HOXB7-Cre; Vhlh* ^*fl/fl*^ *; Hif-2α* ^*fl/fl*^ double knockout) (Vhlh^-/-^; Hif2α^-/-^) serum erythropoietin levels returned to normal, compared with wild-type (WT). *Hif-2α* single allele knockout did not significantly affect the *Vhlh* knockout Epo level (*HOXB7-Cre; Vhlh* ^*fl/fl*^ *; Hif-2α* ^*fl/+*^) (Vhlh^-/-^; Hif2α^+/-^). Data were analyzed by Student’s *t*-test with Welch’s correction for unequal variance, followed by Bonferoni post-test. **c** Erythroid progenitors (TER119 + CD71+) were isolated from hemangiomas of two (labeled 1, 2) knockout mice with FACS (purity ~80 %). Genomic DNA was prepared and used for PCR for the recombined, the deleted *Vhlh* allele *(Vhlh del)*, and the floxed allele (*Vhlh flox*). No deleted allele was detected in these erythrocyte progenitors. As controls, PCR without template (no template) and PCR with genomic DNA from liver of knockout mice (containing both deleted and floxed alleles; positive control) was performed. **d** Hemangiomas were rescued in *HOXB7-Cre; Vhlh* ^*fl/fl*^ *; Hif-2α* ^*fl/fl*^ double knockouts. Hemangiomas were counted in H&E stained liver sections of 2-3 months old mice of indicated genotypes. While the number of hemangiomas per liver section was significantly increased in *Vhlh* knockouts compared to the wild-type, the number of hemangiomas was significantly decreased in *HOXB7-Cre; Vhlh* ^*fl/fl*^ *; Hif-2a* ^*fl/fl*^ double knockout mice compared with *HOXB7-Cre; Vhlh* ^*fl/fl*^ single knockouts. Each data point represents one liver sample. Counts were averaged from at least 5 sections per liver. p-values were calculated using Student’s *t*-test with Welch’s correction. *: statistically significant (*p* < 0.05); ns: not significant

Hemangioblastoma associated with the VHL disease often presents with extramedullary erythropoiesis [17]. Indeed in our model, quantification of erythrocyte progenitors in livers of wild-type and knockout mice by flow cytometry (TER119 + CD71+; pooled data from >2 month old mice; Fig. 2a) indicated that there was a >10-fold increase in erythrocyte progenitors in livers of knockout mice. The extent of increase in TER119 + CD71+ cell number is in agreement with colony forming unit assay that functionally defines erythrocyte progenitors (Fig. 2b). Epo is a key cytokine that promotes erythropoiesis, including the formation of BFU-E and the subsequent differentiation steps to form erythrocytes [30]. Its expression is induced under hypoxic conditions by HIF transcription factor and in *VHL* mutant cells. We indeed demonstrated that the increased number of erythrocyte progenitors correlated with over-expression of Epo: *Epo* mRNA was up-regulated in liver of the knockout mice (Fig. 3a), and Epo protein was increased in the serum (Fig. 3b) in the *Vhlh* knockout mice. However, *Vhlh* deletion is not present in these progenitor cells (Fig. 3c), indicating that a non-erythroid *Vhlh* ^*−*^ component is responsible for inducing extramedullary erythropoiesis.

### Hepatic phenotypes of *HOXB7-Cre* driven *Vhlh* knockout mice are *Hif-2α*-dependent

Previous conditional knockout mice with *Vhlh* inactivation in hepatocytes developed liver hemangiomas and erythrocytosis, but no hemangioma-associated extramedullary erythropoiesis [22, 24]. In those mouse models, hemangiomas and erythrocytosis were rescued by *Hif-2α* inactivation. We therefore examined the effects of *Hif-2α* inactivation on the phenotypes observed in our mouse model by generating *HOXB7-Cre; Vhlh* ^*fl/fl*^ *; Hif-2α* ^*fl/fl*^ double knockouts. *Hif-2α* knockout rescued elevated serum Epo (Fig. 3b) as well as extramedullary erythropoiesis, as assessed with colony forming unit assay (Fig. 2b). *Hif-2α* inactivation also ameliorated the hemangioma phenotype. The onset of hemangiomas was delayed (25 % vs 90 % at 6 weeks), and the number of hemangiomas per liver was significantly reduced in 2 to 3-month old double knockouts (Fig. 3d).

### *Vhlh* inactivation occurs in liver leukocytes, not in liver parenchyma

**Fig. 4:**
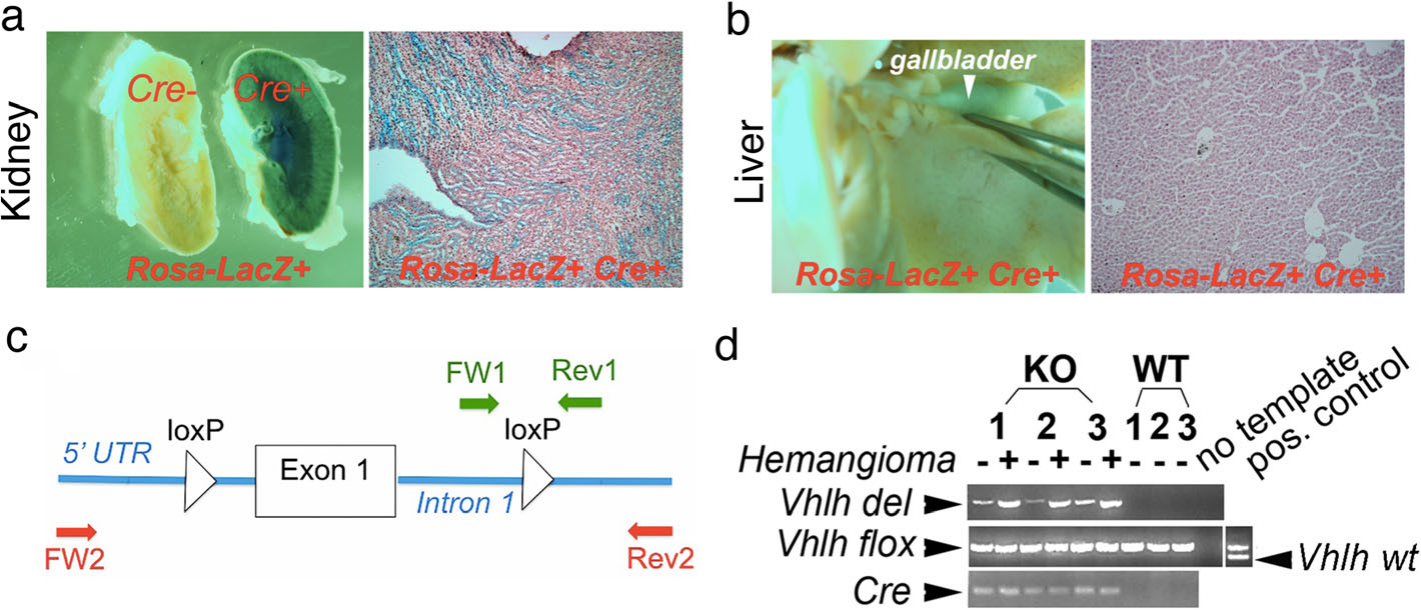
*HOXB7-Cre* mediates deletion in liver leukocytes. **a**, **b** *Rosa-LacZ* reporter detects *HOXB7*-driven *Cre* expression in the kidney, but not in the liver. **a** Kidney of *Rosa-LacZ+* (Cre-) *or HOXB7-Cre; Rosa-LacZ+*(Cre+) was stained with β-galactosidase substrate. **b** Liver of *HOXB7-Cre; Rosa-LacZ+* mouse. Left panels show gross morphological appearance of whole-mount stains (kidneys were sectioned in halves), and right panels show sections prepared from whole-mounts. Strong LacZ signal is seen in medulla and cortex of *HOXB7-Cre; Rosa-LacZ+* kidney (**a**), but not in *Cre* negative *Rosa-LacZ+* kidney (**a**) or *HOXB7Cre+; Rosa-LacZ+* liver (**b**). **c** Map of the floxed *Vhlh* allele (based on description by Haase et al. [20]) and locations of primers for detection of wild-type and floxed *Vhlh* allele (FW1-Rev1, green; primers designed by Rankin et al. [22]) and recombined, deleted allele (FW2-Rev2, red; primers design is as described in Methods). Not drawn to scale. FW, forward primer; Rev, reverse primer; 5′UTR, 5 prime untranslated region; loxP, loxP site. **d** By PCR, *Vhlh* deletion is detected in livers of *HOXB7-Cre; Vhlh* ^*fl/fl*^ mice. Genomic DNA was isolated from livers of *HOXB7-Cre; Vhlh* ^*fl/fl*^ (KO) mice and *Cre* negative littermates (WT). For each genotype, genomic DNA from three different mice (1–3) was obtained. PCR was performed with primers specific for the wild-type (*Vhlh wt*), floxed (*Vhlh flox*) or recombined (deleted) (*Vhlh del*) alleles using primers described in **c**. In addition, *Cre*-specific PCR was performed. Knockout DNA samples were isolated from either liver tissue with healthy appearance (-) or liver tissue with hemangiomas (+). Note that the recombined *Vhlh* allele (*Vhlh del*) was only detected in knockout mice, confirming the specificity of PCR. Furthermore, the signal for the recombined allele was much stronger in knockout liver tissue with hemangiomas (+) compared to knockout liver tissue with healthy appearance (-). As controls, PCR was performed without template (no template) or with liver DNA from a *Vhlh* ^*fl/+*^ mouse (positive control for floxed and wild-type *Vhlh* alleles)

Although the hemangioma phenotype recalled the outcome of previous hepatocyte-specific *Vhlh* knockout mice [20, 22], to our knowledge, the *HOXB7-Cre* driver used in our study has not been shown to function in the liver. We therefore used the *ROSA-LacZ* reporter [31] to determine whether this *Cre* driver mediated gene inactivation in the hepatocytes. As previously shown, Cre activity was readily detected in cortex and medulla of kidneys from *HOXB7-Cre*; *Rosa-LacZ+* mice, whereas no Cre activity was detected in kidneys of *Cre-*negative *ROSA-LacZ+* mice (Fig. 4a, left panel, compare *Cre-* and *Cre +* kidney) [25, 28, 29]. In contrast, we observed no overt Cre activity in the liver of *HOXB7-Cre*; *ROSA-LacZ+* mice (Fig. 4b). We also did not observe steatosis in hepatocytes, a phenotype characteristic of *Vhlh* null hepatocytes [20, 22] (data not shown). It should be noted that the *ROSA-LacZ* locus is on the same chromosome as the *Vhlh* locus; therefore the reporter could not be used to trace *Vhlh* knockout cells. When an alternative and more sensitive PCR method was used, *Vhlh* inactivation was detectable in the livers of knockout mice. We used previously published primers to detect the wild-type and floxed *Vhlh* alleles [22], and designed new primers to detect the recombined (deleted) *Vhlh* allele (see Methods and Fig. 4c). Using these primers, we were able to detect the recombined *Vhlh* allele (*Vhlh* ^*del*^) in the livers of *HOXB7-Cre; Vhlh* ^*fl/fl*^ mice (Fig. 4d, lanes labeled with KO). Confirming the specificity of the primers, no deletion was detected in the livers of *Cre-*negative *Vhlh* ^*fl/fl*^ littermates (Fig. 4d, lanes labeled with WT). We also confirmed the identity of the *Vhlh* ^*del*^ band by sequencing (data not shown). Next, both hemangiomas and gross-morphologically healthy looking liver tissue were dissected out of livers of *HOXB7-Cre; Vhlh* ^*fl/fl*^ mice. Interestingly, a much stronger signal for the deleted allele was observed in hemangioma tissue compared to adjacent normal liver tissue [Fig. 4d, compare lanes labeled – (no hemangioma) and + (with hemangioma)], indicating that *Vhlh* null cells were enriched in the hemangiomas.

**Fig. 5:**
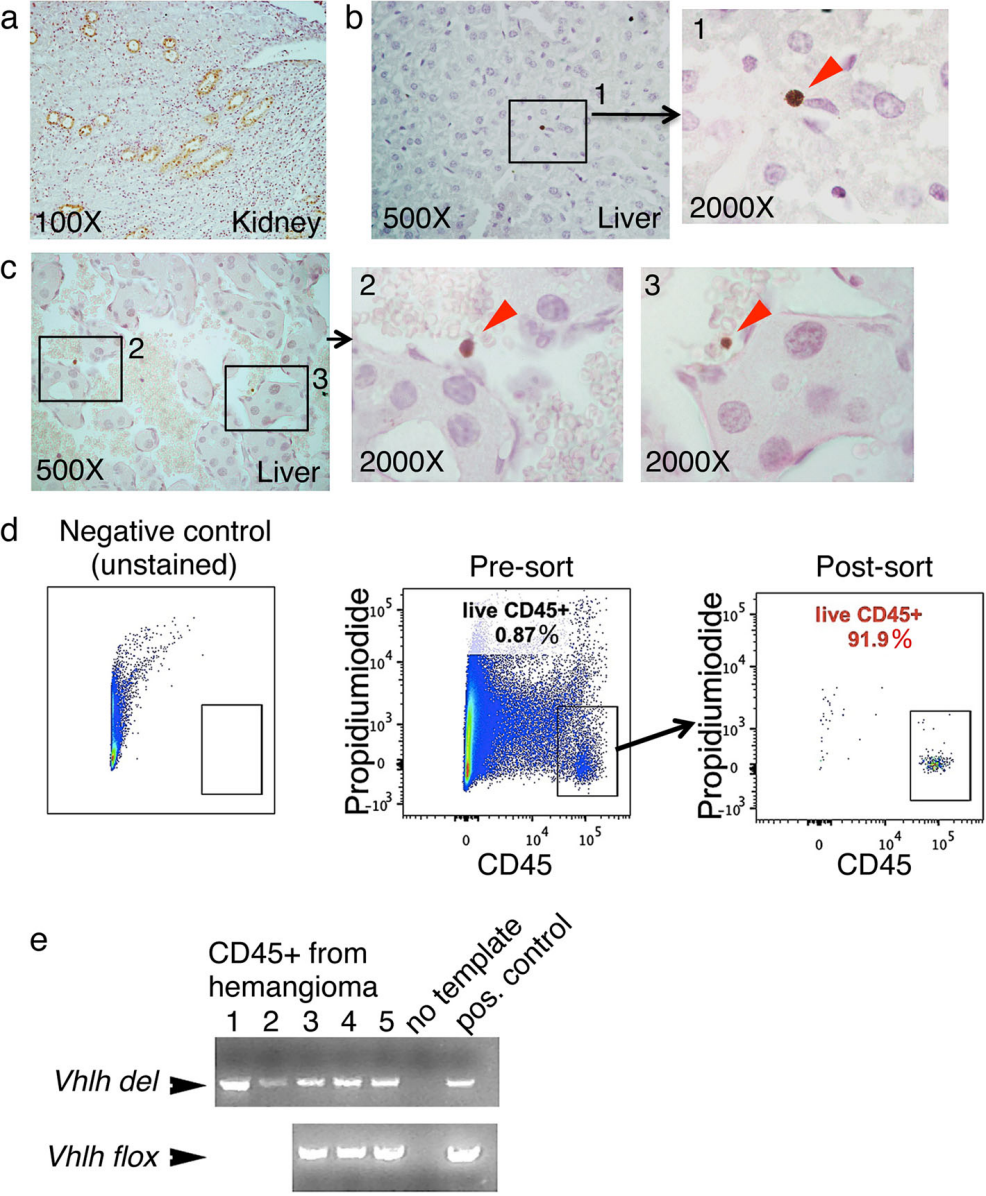
Deletion of *Vhlh* in liver leukocytes. **a** By immunohistochemistry, GFP is prominent in the collecting ducts in kidney of the reporter strain *HOXB7-GFP-Cre; Vhlh* ^*fl/fl*^ [25, 29]. **b**, **c** GFP is also detectable in single cells interspersed within the liver parenchyma (**b1**) or within hemangiomas (**c2**, **3**). **d** Isolation of leukocytes from livers of *HOXB7-Cre; Vhlh* ^*fl/fl*^ mice. Liver cell suspensions were prepared by mincing livers, digesting with collagenase, and lysing red blood cells with red blood cell lysis buffer. Liver cell suspensions were then stained with the pan-leukocyte marker CD45 and propidium iodide (live/dead stain). Live CD45+ cells were isolated with FACS; debris and doublets were gated out. After the sort, CD45+ fractions were >90 % pure. **e** By PCR, *Vhlh* inactivation was detected in CD45+ leukocyte fraction of 5 independent samples (1–5). Samples 3–5 were additionally examined for the presence of floxed alleles (the amount of DNA samples 1 and 2 were insufficient for additional PCR tests). As control, PCR was performed without template (no template control) or with genomic DNA from a *HOXB7-Cre; Vhlh* ^*fl/fl*^ knockout kidney (positive control, which contains both *Vhlh*-inactivated and wild-type cells)

The lack of Cre + cells in wild-type liver, and enrichment of *Vhlh* deletion allele in the hemangiomas of the knockouts raised the possibility that *Vhlh* deletion occurred in an invading or locally expanded cell population. To determine in which cell type *Vhlh* was inactivated, we used another *Cre*-reporter: the *HOXB7-GFP-Cre* driver that can be combined with the *Vhlh* flox allele. In the *HOXB7-GFP-Cre* driver, *Gfp* and *Cre* are made from a bicistronic message, and GFP is readily detectable in the kidney {Fig. 5a and [25, 29]}. Using this reporter, we detected GFP expression in isolated, non-hepatic cells interspersed in the liver parenchyma, and within the hemangiomas (Fig. 5b, c). These cells appeared to be leukocytes. Indeed, by PCR, *Vhlh* inactivation was detectable in leukocytes (CD45+ fraction) isolated from hemangiomas by FACS (Fig. 5d, e). The unrecombined floxed allele was also detectable in this fraction (Fig. 5e), which is not surprising since these leukocytes are heterogeneous, comprising B cells, T cells, NK cells and myeloid cells (data not shown). Of note, GFP is not detected in the endothelia (Fig. 5b, c), and as shown above (Fig. 2d), the erythrocyte progenitors isolated from the liver of knockout mice did not contain *Vhlh* deletion allele, either. We therefore conclude that in this mouse model, *Vhlh* is inactivated in a subset of liver leukocytes.

### *Vhlh* inactivation is detected in granulocytes/neutrophils

**Fig. 6:**
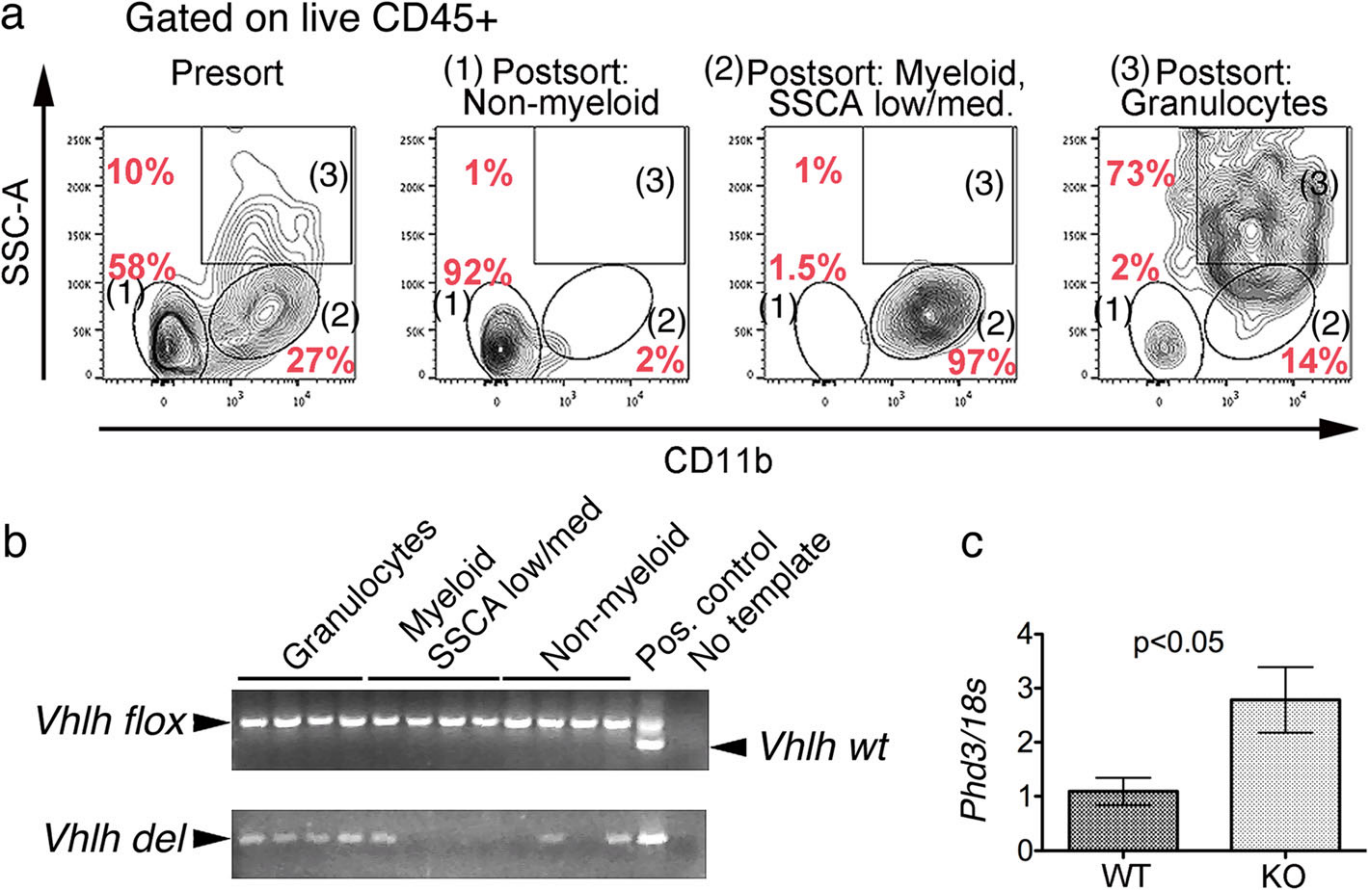
*Vhlh* is deleted in granulocytes of *HOXB7-Cre; Vhlh* ^*fl/fl*^ mice. **a** Isolation of (1) non-myeloid cells (CD11b-), (2) other myeloid cells (CD11b + SSCA low/medium), and (3) granulocytes (CD11b + SSCA high) from *HOXB7-Cre; Vhlh* ^*fl/fl*^ mice by FACS. Shown are representative FACS-plots before and after sorting. The three populations are indicated in each panel. **b** PCR analysis of *Vhlh* alleles in genomic DNAs isolated from the above cell populations. Recombined *Vhlh* allele (*Vhlh del*) is consistently observed in (3) granulocyte-enriched fraction (~95 % CD11b+, 70 % CD11b + SSCA high). As control, PCR was performed without template (no template control) or with genomic DNA from the liver of a *HOXB7-Cre; Vhlh* ^*fl/+*^ mouse (positive control containing *Vhlh*-floxed and wild-type cells) or a *HOXB7-Cre; Vhlh* ^*fl/fl*^ kidney (positive control containing *Vhlh*-del cells). **c** Consistent with *Vhlh* inactivation, the mRNA of *Proline-hydroxylase 3* (*Phd3*), a Hif-2α-responsive gene, is up-regulated in the granulocyte fraction, as assayed by qPCR

*Hoxb7* expression has been observed in granulocytes isolated from murine bone marrow [32]. This would agree with our observation of *Vhlh* inactivation in leukocytes. We therefore isolated granulocytes (CD11b + SSC-A high) from liver hemangiomas by FACS (Fig. 6a) and were able to detect *Vhlh* deletion consistently in the granulocyte-enriched fraction (Fig. 6b). *Vhlh* inactivation was also occasionally observed in the CD11b + SSC-A medium/low fraction, a fraction that contains some granulocytes besides other myeloid cells; and in the non-myeloid fraction (Fig. 6b). The appearance of *Vhlh* deletion in these non-granulocyte fractions is inconsistent; it is therefore difficult to assess their functional significance. Consistent with *Vhlh* inactivation, we also observed significant up-regulation of the *Hif-2α* responsive gene *Phd3* in granulocytes (Fig. 6c). Taken together, these data indicate that the *HOXB7-Cre* driver mediates inactivation in a subset of granulocytes.

**Fig. 7:**
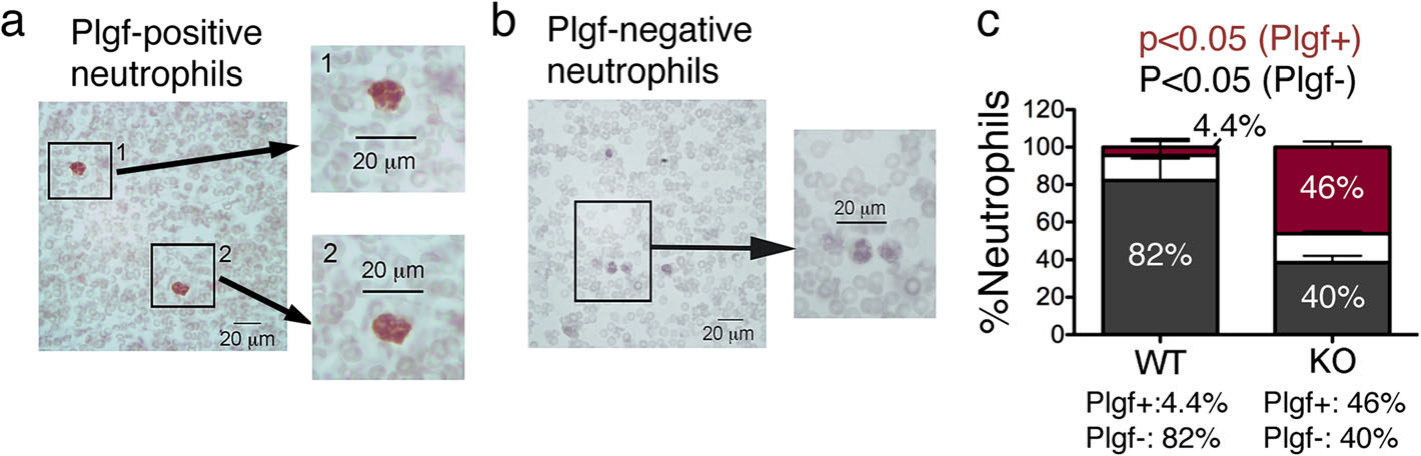
Plgf is expressed in neutrophils of *HOXB7-Cre; Vhlh* ^*fl/fl*^ knockout mice. **a**, **b** Plgf immunohistochemistry of liver sections (brown stain). Nuclei were stained with hematoxylin (light blue). Representatives Plgf + (**a**) and Plgf- (**b**) neutrophils localized within hemangiomas of a *HOXB7-Cre; Vhlh* ^*fl/fl*^ knockout mouse are shown. Note the polymorpho-nuclear shape characteristic of neutrophils. **c** Quantification of Plgf-expressing liver neutrophils (immunohistochemistry); *n* = 3 mice per group, total number of neutrophils counted: 38 (WT) and 510 (KO). Neutrophils were classified as strongly Plgf positive (Plgf+, dark pink), weakly Plgf positive (Plgf+/-, white) or Plgf negative (Plgf-, grey). A ~10-fold increase in the percentage of Plgf + neutrophils was observed in *HOXB7-Cre; Vhlh* ^*fl/fl*^ mice compared to *Cre*-negative littermates (46 % versus 4.4 %)

Among the HIF-dependent pro-angiogenic factors, we have detected increased expression of vascular endothelial growth factor B (VEGFB) and placental growth factor (Plgf) in the liver of knockout mice (data not shown). We used Plgf as a marker to examine the cell type potentially contributing to the liver phenotype. As shown in Fig. 7, both Plgf-positive and negative neutrophils could be observed (Fig. 7a, b). Interestingly, the percentage of Plgf-positive neutrophils was significantly increased (>10 folds) in the liver of knockout mice compared with the wild-type (Fig. 7c).

### Hepatic phenotypes were rescued by reconstitution with wild-type hematopoietic stem cells

**Fig. 8:**
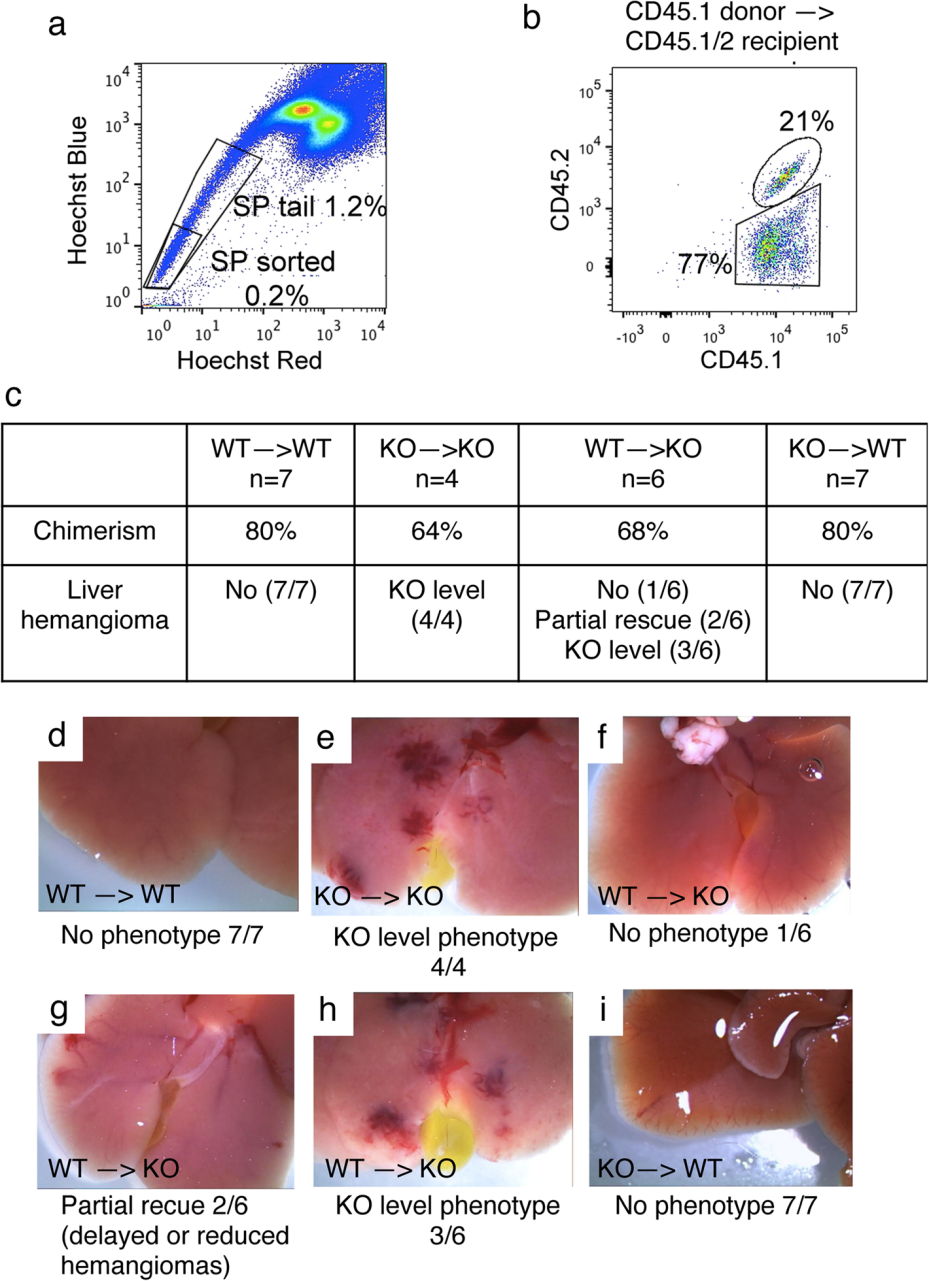
Hemangiomas in knockout mice are partially rescued by reconstitution with wild-type hematopoietic stem cells. Hematopoietic stem cells were enriched by sorting Hoechst negative side population (SP) from bone marrow. 8–9-week old wild-type mice or 4-week old *HOXB7-Cre; Vhlh* ^*fl/fl*^ mice were lethally irradiated and transplanted with 1000 SP obtained from 2 to 3 month old *HOXB7-Cre; Vhlh* ^*fl/fl*^ mice (KO), or *Cre-*negative *Vhlh* ^*fl/fl*^ littermates (WT). **a** Representative Hoechst stain followed by sorting. Percentage of Hoechst negative cells is indicated. Only the tip of the Hoechst negative tail that contains cells with the least Hoechst fluorescence was collected. **b** Representative flow data of peripheral blood leukocytes stained with CD45.1 and CD45.2 two months after the transplant (wild-type recipients) or one month after the transplant (*HOXB7-Cre; Vhlh* ^*fl/fl*^ recipients). Recipients were CD45.1/2 double positive and were transplanted with CD45.1 single positive SP. **c** Summary of transplant experiments. **d**–**i** Representative phenotypes of the transplanted chimera. Livers of KO recipients were obtained one month after the transplant, at ~2 months of age; livers of WT recipients were obtained at 2, 3 and 6 months after transplantation. Numbers beneath images indicate number of mice with phenotype versus total number of mice analyzed. **d** Wild-type SP to wild-type recipients (WT - > WT) did not show any phenotype. **e** Knockout SP to knockout recipients (KO - > KO) showed full-penetrant phenotype. **f**–**h** Hemangiomas are partially rescued in *HOXB7-Cre; Vhlh* ^*fl/fl*^ recipient mice (KO) transplanted with SP from *Cre negative Vhlh* ^*fl/fl*^ mice (WT). **i** Knockout SP to wild-type recipients (KO - > WT) showed no phenotype

To elucidate the contribution of *Vhlh* null granulocytes to hemangioma formation, we generated bone marrow chimeras. Hematopoietic stem cells were enriched by sorting the Hoechst-negative “side population” (SP) from bone marrow (Fig. 8a) [26]. Lethally irradiated mice were reconstituted with 1000 SP cells, and the chimerism—defined as percentage of peripheral blood leukocytes derived from donor stem cells—was determined one month (knockout recipients) or 2–6 months (wild-type recipients) after transplantation to confirm the success of the transplant (Fig. 8b). A chimerism of 60–80 % was obtained (Fig. 8c). Control experiments showed that wild-type donor to wild-type recipient transplantation did not cause any liver phenotype (Fig. 8c, d); and that knockout donor to knockout recipient transplantation generated liver hemangioma phenotype with severity indistinguishable from the *HOXB7-Cre; Vhlh* ^*fl/fl*^ mice (Fig. 8c, e). In the wild-type to knockout chimeric mice, hemangiomas were rescued (Fig. 8c, f) or improved (Fig. 8c, g) in 50 % of knockout mice (Fig. 8c, h). The partial rescue could be because the replacement of the host hematopoietic stem cells (chimerism) was not complete. However, it is also possible that other non-bone marrow-derived components might be involved. Interestingly, knockout to wild-type transplantation did not generate the hemangioma phenotype, despite high chimerism (Fig. 8c, i). This indicates that *Vhlh* inactivation in the hematopoietic component is necessary but insufficient for the hemangioma formation.

## Discussion

The kidney phenotype in *HOXB7-Cre; Vhlh* ^*fl/fl*^ knockout mice develops at ~2 months of age {[25] and data not shown}. In contrast, nascent hemangiomas are already apparent in 75 % of 4-week old mice, and well-developed hemangiomas are present in 90 % of 6-week old mice (Fig. 1d). Furthermore, *Hif-2α* inactivation ameliorates the hemangioma phenotype, but has no effect on the kidney phenotype. Thus, the liver phenotype is most likely not the result of *Vhlh* inactivation in the kidney lesion. We cannot exclude the possibility that Hif-2α-induced factors emanating from the kidney are required for liver hemangioma formation. However, this explanation is not favored because other kidney-specific *Vhlh* knockouts do not develop liver phenotype [11, 33].

Liver hemangiomas have been described previously in germline *Vhlh* ^*+/-*^ mice and in mice with *Vhlh* inactivation in hepatocytes (*PEPCK-Cre* or *Albumin-Cre* driven *Vhlh* knockout mice) [20, 22, 23]. *PEPCK*-driven *Cre* is worth noting because it has dual specificity in kidney (proximal tubules) and liver (~20–30 % hepatocytes) [22]. Here, we show that *HOXB7-Cre driven Vhlh* conditional knockout mice also develop hemangiomas in the liver, although unlike previous models, this *Cre* driver does not mediate recombination in hepatocytes. We were unable to detect Cre activity in hepatocytes using the *Rosa-LacZ* reporter, and we also did not observe steatosis in hepatocytes, a phenotype characteristic of *Vhlh* null hepatocytes [20, 22].

The more hepatocyte-specific *Cre* (*Albumin-Cre*) driven *Vhlh* knockout also generated hemangiomas, albeit more at the microscopic level [22]. However, despite superficial similarity of the phenotypes, several findings indicate that the mechanism of hemangioma formation is different in hepatocyte-specific *Albumin-Cre* driven and *HOXB7-Cre* driven *Vhlh* knockout mice. In *HOXB7-Cre* driven *Vhlh* knockout mice, hemangiomas develop with higher penetrance (90 % vs ~40 %) and at a younger age (4–6 weeks versus ~3–8 months) compared to the *Albumin-Cre Vhlh* knockout mice [20]. Furthermore, the hemangiomas in *HOXB7-Cre* driven *Vhlh* knockouts exhibit extramedullary hematopoiesis that is also observed in human hemangioblastoma but not in liver with hepatocyte *Vhlh* knockout (either *Albumin-Cre* or *PEPCK-Cre* driven).

Another difference between the hepatocyte-specific *Vhlh* knockout mice and the *HOXB7-Cre* driven *Vhlh* knockout mice is the pattern of erythropoiesis. Erythropoietin is over-expressed in the knockout mice with hepatocyte-specific *Vhlh* inactivation [20, 22], as well as in *HOXB7-Cre* driven *Vhlh* knockout mice. However, while erythropoietin over-expression induced systemic erythrocytosis—characterized by elevated hematocrit—in *PEPCK-Cre* and *Albumin-Cre* driven *Vhlh* knockout mice [20, 22], erythropoiesis was only increased in *HOXB7-Cre* driven *Vhlh* knockout mice in specific organs such as liver. There is no increase in hematocrit in *HOXB7-Cre* driven *Vhlh* knockouts (data not shown). This pattern of focal erythropoiesis resembles the pattern observed in VHL patients, who develop polycythemia only rarely, despite tumor-associated extramedullary erythropoiesis [34].

By PCR, a stronger signal for the recombined allele was detected in hemangiomas compared to healthy liver tissue, indicating that *Vhlh* null cells are enriched in hemangiomas. Also, Cre activity was undetectable with the *Rosa-LacZ* reporter in mice with wild-type *Vhlh*. Thus, *Vhlh* inactivation likely occurs in a cell type that is rare in the healthy liver and undergoes expansion or is recruited to the liver when *Vhlh* is inactivated.

One type of cells that was enriched in hemangiomas is leukocytes. We detected foci of leukocytes (CD45+) adjacent to hemangiomas, and by PCR we detected *Vhlh* inactivation in leukocytes isolated from hemangiomas (Fig. 5). We further identified granulocytes/neutrophils as the *Vhlh* mutant cells in this model (Figs. 6 and 7). Furthermore, hemangiomas were partially rescued in *HOXB7-Cre; Vhlh* ^*fl/fl*^ mice reconstituted with wild-type hematopoietic stem cells (Fig. 8). Thus, *Vhlh* inactivation in bone marrow-derived leukocytes contributes to hemangioma formation in this mouse model. Interestingly, *Vhlh* null leukocytes were not sufficient to induce hemangiomas, since no hemangiomas were observed in the reverse experiment (wild-type mice reconstituted with *HOXB7-Cre; Vhlh* ^*fl/fl*^ hematopoietic stem cells). Thus, inactivation of *Vhlh* in at least one other cell type besides bone marrow-derived cells is required for hemangioma formation. It may be that *HOXB7-Cre* driven *Vhlh* knockout in kidney tubule cells contributes to systemic elevation of Epo, which in turn is required for hemangioma formation in the liver. However, kidney hyperplasia and tubule defects were rescued by inactivation of *Hif-1α*, but not *Hif-2α* [25], whereas other kidney-specific *Vhlh* knockout mouse models did not develop liver hemangioma [11, 33]. The other contributing cell types might include embryonic hematopoietic progenitor cells in the liver that fail to migrate to the bone marrow. However, we did not detect *Vhlh* deletion in the erythrocyte progenitor cells, despite increased number of these progenitors. We did, however, detect *Vhlh* deletion in liver granulocytes/neutrophils and moderately increased number of Granulocyte/Macrophage/Megakaryocyte/Erythroid (GMME) progenitors cells in the liver (data not shown). This indicates that *Vhlh* mutant liver-resident granulocyte progenitors may be critical for liver extramedullary erythropoiesis and hemangioma formation. Interestingly, it has been reported that *VHL* haploid insufficiency in neutrophils contributes to their decreased apoptosis, and thus population expansion, in VHL patients. It may be that liver resident granulocytes/neutrophils undergo expansion and promote angiogenesis. We indeed observed increased population of Plgf-expressing neutrophils in knockout mice compared to wild-type. Therefore both bone marrow-derived and liver-resident granulocytes/neutrophils are needed for the full penetrance of liver hemangioma phenotype.

We have not observed the hemangioma phenotype in other organs of the *Vhlh* knockout mice described here. This indicates that a unique hepatic microenvironment may be required. However, since the kidney defects observed in the *Hoxb7-Cre; Vhlh* ^*fl/fl*^ knockout mice include hyper-vascularization and hemorrhage [25], it is tempting to speculate that the activated neutrophils may also contribute to the full extent of the kidney hyperplastic phenotypes. Future studies should address this intriguing possibility.

The finding that *Vhlh* null granulocytes contribute to hemangioma formation is of significance for understanding hemangioblastoma and other highly vascularized VHL tumors. Since VHL patients are heterozygous for *VHL* loss-of-function mutations, it is conceivable that inactivation of the remaining wild-type allele could occur in more than one cell type (as indeed it does, since patients are prone to develop tumors in several tissues), including those constituting the stromal compartments. Mutations in the cancer stromal cells have been documented that contribute to cancer progression {reviewed in [12]}. In particular, neutrophils have been recognized as a major inducer of tumor angiogenesis [35]. Significantly, recent reports have identified CXCR4 (a known HIF target)-expressing neutrophils as a highly pro-angiogenic subtype of neutrophils [36]. It is therefore possible that in VHL patients, *VHL* inactivation occurs in components of the tumor microenvironment in addition to the tumor itself. Pro-angiogenic *VHL* null granulocytes/neutrophils could therefore contribute to the development of the tumor vasculature.

## Conclusions

Hemangioblastoma is a serious tumor associated with the VHL disease and is difficult to study because of a lack of animal models. One interesting pathological feature of this disorder is that the endothelial cells are themselves genomically normal. Instead, vascular overgrowth is induced by adjacent non-endothelial cells that exhibit loss of heterozygosity and may be of hematopoietic or embryonic origin. Our mouse model shows that *Vhlh* inactivation in the “stromal” (non-tumor) cell population, at least in part consisting of granulocytes/neutrophils, can induce abnormal angiogenesis. Furthermore, we show that hemangioma-associated extramedullary erythropoiesis can occur without *Vhlh* inactivation taking place in erythrocytes. This system thus represents a good model for studying aspects of the hemangioblastoma found in the human VHL disease.

## Abbreviations

ARNT: Aryl hydrocarbon receptor nuclear translocator
ccRCC: Clear-cell renal cell carcinoma
Epo: Erythropoietin
FACS: Fluorescence-activated cell sorting
HIF: Hypoxia-inducible factor
PCR: Polymerase chain reaction
Plgf: Placental growth factor
VEGF: Vascular endothelial growth factor
VHL: von Hippel-Lindau
